# Control of blood pressure in hypertensive children and adolescents assessed by ambulatory blood pressure monitoring

**DOI:** 10.1186/s12887-024-04732-z

**Published:** 2024-04-25

**Authors:** Kevalin Vigraijaroenying, Kwanchai Pirojsakul, Poomiporn Katanyuwong, Kanchana Tangnararatchakit, Pawaree Saisawat, Songkiat Chantarogh, Witchuri Paksi, Uthen Bunmee

**Affiliations:** Division of Nephrology, Department of Pediatrics, Faculty of Medicine Ramathibodi Hospital, Mahidol University, Bangkok, Thailand

**Keywords:** Control of blood pressure, Children, Adolescents, Ambulatory blood pressure monitoring

## Abstract

**Background:**

There have been few studies evaluating the control of hypertension (HT) in children. This study aimed to assess the control of HT using ambulatory blood pressure monitoring (ABPM) and to compare the parameters between the uncontrolled HT and controlled HT groups.

**Methods:**

Hypertensive patients aged ≥ 5 years who underwent ABPM to assess the control of HT were enrolled. Demographics, office blood pressure (BP), ABPM, and echocardiographic data were collected. Controlled HT was defined using a BP goal recommended by the 2016 European Society of Hypertension guidelines.

**Results:**

There were 108 patients (64.8% males) with a mean age of 14.3 years and 51.9% had primary HT. Controlled HT was detected in 41.1% and 33.3% by office BP and ABPM, respectively. Based on ABPM, there was a greater prevalence of controlled HT in the primary HT than the secondary HT group (44.6% vs. 21.2%, *P* = 0.01). In the primary HT group, BMI z-score at the last follow-up had a significant decrease in the controlled HT than the uncontrolled HT group (-0.39 vs. 0.01, *P* = 0.032). Primary HT was negatively associated with uncontrolled HT by ABPM. In addition, ABPM showed greater sensitivity (77.8% vs. 55.8%) and negative predictive value (80.9% vs. 70.8%) to predict LVH than those of office BP measurement.

**Conclusion:**

Only one-third of patients achieved the BP goal by ABPM and most were in the primary HT group. Weight reduction is an important measure of BP control in patients with primary HT to attenuate the risk of LVH.

**Supplementary Information:**

The online version contains supplementary material available at 10.1186/s12887-024-04732-z.

## Introduction

Hypertension (HT) has been a common cardiovascular condition seen in children and adolescents over the last decade. The causes of HT include: (1) primary HT, seen mainly in patients older than 6 years of age with overweight or obesity or having a family history of HT, (2) secondary HT, seen in patients with kidney diseases, renovascular diseases, endocrinologic diseases, neurologic diseases, and using medications affecting blood pressure (BP) [1–3]. According to the American Academy of Pediatrics’ (AAP) 2017 recommendations, HT should be diagnosed based on three instances of consistently high office BP[1]. In Thailand, most pediatric nephrologists currently use the 2017 AAP recommendations as the guidelines for diagnosis and management of HT in children and adolescents as they are scientific evidence-based recommendations and are simple to use for diagnosis of HT by office BP measurement.

Ambulatory blood pressure monitoring (ABPM) has been increasingly used in children and adolescents over the past decades. It is a valuable procedure used not only to confirm the diagnosis of HT but also to evaluate the control of BP in children diagnosed with HT [1]. The advantages of ABPM over office BP measurements used in children with HT are the ability to reveal masked uncontrolled HT (a condition with controlled office BP but uncontrolled ABPM) and white coat effect (a condition with uncontrolled office BP but controlled ABPM). These benefits can help pediatricians correctly adjust anti-hypertensive medications and may help early detect the complication of HT.

The 2016 European Society of Hypertension (ESH) guidelines recommended the BP goal for both office BP measurement and ABPM in treated hypertensive children based on the classification of HT and co-morbidities such as chronic kidney disease, proteinuria, and diabetes mellitus. The 2016 ESH guidelines provided the BP goals for hypertensive children with different settings such as primary HT, HT with diabetes mellitus, HT with chronic kidney disease and HT with or without proteinuria [3]. The different BP goals for each patient would help guiding the pediatricians to practice individualized medicine for hypertensive children as BP control in hypertensive children and adolescents is also crucial to prevent cardiovascular sequelae, kidney damage in individuals with normal kidneys and deterioration of kidney function in patients with existing chronic kidney disease [3].

In the previous studies, the control of HT was adequate in 22–57% of patients with various causes of HT [4–8]. However, there have been a few studies that focus on the control of HT in pediatric patients assessed by ABPM. Some studies found that an inadequate BP control was associated with a greater prevalence of left ventricular hypertrophy (LVH) [4, 9, 10]. However, no study had defined any parameters associated with the control of HT and the long-term outcomes. The present study aimed to assess the control of HT using ABPM and to compare the parameters between the uncontrolled HT and controlled HT groups in children and adolescents.

## Materials and methods

### Subjects

The present study retrospectively included hypertensive patients aged ≥ 5 years who had been diagnosed with HT by office BP measurement based on the recommendation of the 2017 AAP [1] and subsequently underwent ABPM for assessment of BP control. A total of 115 patients performed ABPM after the diagnosis of hypertension were eligible. Seven patients were excluded due to chronic kidney disease stage 5 and were receiving kidney replacement therapy. The remaining 108 patients were included for the final analysis. HT was classified into 2 groups including primary and secondary HT. The present study was approved by the Ethics Committee for Human Research, Faculty of Medicine Ramathibodi Hospital, Mahidol University (MURA2022/88).

### Data collection

Data at the time of diagnosis and at the first ABPM for follow-up after treatment were collected including age, sex, classification of HT, body mass index (BMI) z-score, office BP measurement, estimated glomerular filtration rate (eGFR) calculated by age- and sex-dependent under 25 (U25) GFR estimating equations [11], proteinuria (defined as spot urine protein/urine creatinine ratio (UPCR) ≥ 0.2 mg/mg) and anti-hypertensive medications used. Overweight was defined as BMI z-score between 1 and 1.99 and obesity was defined as BMI-for-age z-score ≥ 2 by using the WHO Anthroplus software.

Office BP was measured twice by a Dinamap Pro care (GE Healthcare, Chicago) in the right arm after resting for 5 min. Appropriate BP cuff size for each patient was used as recommended by the current guidelines [12]. The office BP used in the analysis was an average of the two measurements and was classified according to the 2017 AAP guidelines [1]. The office BP goal used in the present study followed the recommendations of the 2016 ESH guidelines for the management of high BP in children and adolescents [3]. An office systolic BP (SBP) or diastolic BP (DBP) index for each patient was calculated by patient’s SBP or DBP divided by patient’s office SBP or DBP goal. Therefore, an uncontrolled HT by office BP measurement was diagnosed in a patient who had a SBP or DBP index ≥ 1.

The ABPM device used in the present study was a TM-2430 device (A&D, Tokyo) which has been validated for use in children and adolescents [13]. A BP cuff was applied using an appropriately-sized cuff and placed on the non-dominant arm for each patient. The device was programmed to record BP for a 24-hour period by measuring every 20 min during awake period and every 30 min during sleep period based on each patient’s sleep diary. Patients were instructed to perform their routine activities and avoid strenuous activities. The ABPM study had to meet the criteria of at least 40 valid BP readings over a 24-hour period. ABPM parameters included mean SBP and DBP for daytime, nighttime, and 24 h-period.

An ABPM SBP or DBP index for each patient was calculated by patient’s mean SBP or DBP divided by patient’s ABPM SBP or DBP goal for daytime, nighttime and a 24-hour periods. Therefore, an uncontrolled HT by ABPM was diagnosed in a patient who had either a daytime, nighttime, and 24-hour ABPM SBP or DBP index ≥ 1. The percentage of nocturnal BP dipping was calculated as follows: [(mean daytime - mean nighttime)/ mean daytime] x 100 for both SBP and DBP. Masked uncontrolled HT was defined as controlled HT by office BP measurement but uncontrolled HT by ABPM. The white coat effect was defined as uncontrolled HT by office BP measurement but controlled HT by ABPM. The ABPM BP goal used in the present study followed the recommendation of the 2016 ESH guidelines for the management of high BP in children and adolescents [3].

Echocardiography was performed by an experienced pediatric cardiologist and/or a pediatric echocardiographic technician. Left ventricular hypertrophy (LVH) was defined as a left ventricular mass index (LVMI) > 115 g/m^2^ for males and LVMI > 95 g/m^2^ for females [1].

### Statistical analysis

IBM SPSS ® software version 29 was used for statistical analysis. The distribution of each parameter was tested with the Kolmogorov-Smirnov test. Continuous data were expressed as mean (standard deviation, SD) or median (inter-quartile range, IQR) as appropriate. Comparisons between the two groups were tested with the student’s t-test or Mann-Whitney-U test. Categorical data were expressed as numbers and percentages and the comparisons between the two or three groups were analyzed by Pearson’s chi-square test. Logistic regression analysis was used to evaluate parameters associated with uncontrolled HT. A significance level of data analysis was set at *P*-value < 0.05.

## Results

A total of 108 hypertensive patients (70 males, 64.8%) with a mean age of 14.3 ± 3.8 years were enrolled. Patient characteristics at the diagnosis and at the follow-up visit are summarized in Table 1. Fifty-six (51.8%) patients had primary HT while fifty-two patients had secondary HT (44 patients had kidney diseases and 8 patients had non-kidney diseases). Ninety patients received anti-hypertensive medications consisting of angiotensin-converting enzyme inhibitors (ACEI), angiotensin receptor blocker (ARB), calcium-channel blocker (CCB), alpha-blocker and vasodilator. The mean number of anti-hypertensive medications used per patient was 1.5 ± 0.7. There were 37 patients receiving CCB monotherapy, 13 patients using ACEI monotherapy, and 2 patients using ARB monotherapy.

**Table 1:** Demographic data at diagnosis and follow-up in hypertensive children with controlled and uncontrolled HT assessed by ABPM

Parameters	All patients (*N* = 108)	Controlled HT (*N* = 36)	Uncontrolled HT (*N* = 72)	*P value*
**At diagnosis**				
Age, y (mean ± SD)	11.5 ± 4.14	11.17 ± 4.59	11.7 ± 3.91	0.52
Male, N (%)	70 (64.8)	24 (66.7)	46 (63.9)	0.83
BMI z-score, median (IQR)	1.13	2.11	0.64	0.06
	(-0.43, 2.58)	(0.28, 2.76)	(-0.56, 2.5)	
Office SBP index, median (IQR)	1.04 (1, 1.09)	1.04 (1.01, 1.09)	1.03 (1, 1.09)	0.59
Office DBP index, median (IQR)	1 (0.91, 1.08)	1 (0.91, 1.09)	1 (0.92, 1.07)	0.61
Diagnosis by ABPM, N (%)	21 (19.4)	11 (15.3%)	11 (27.8)	0.13
Classification of HT				
- Primary HT (%)	56 (51.85)	25 (69.44)	31 (43.06)	0.006*
- Kidney (%)	44 (40.74)	7 (19.44)	37 (51.39)	
- Non-kidney (%)	8 (7.41)	4 (11.11)	4 (5.56)	
eGFR (ml/min/1.73m^2^), mean ± SD	91.1 ± 50.47	104.15 ± 42.3	84.38 ± 53.24	0.03*
**At Follow-up**				
Age, y (mean ± SD)	14.25 ± 3.81	13.73 ± 3.87	14.5 ± 3.79	0.33
The 1st ABPM after treating HT (y), median (IQR)	1.8 (0.7, 3.7)	2.15 (0.7, 3.5)	1.7 (0.7, 3.9)	0.84
BMI z score, mean ± SD	1.08 ± 1.86	1.54 ± 1.44	0.84 ± 2	0.039*
BMI z score change, median (IQR)	0	-0.22	0.01	0.62
	(-0.54, 0.47)	(-0.58, 0.42)	(-0.52, 0.56)	
24-hr ABPM SBP index, mean ± SD	0.99 ± 0.08	0.92 ± 0.04	1.04 ± 0.07	< 0.001
24-hr ABPM DBP index,	0.93	0.85	0.97	< 0.001
median (IQR)	(0.86, 1)	(0.82, 0.9)	(0.92, 1.05)	
eGFR (ml/min/1.73m^2^), median (IQR)	*N* = 101	*N* = 30	*N* = 71	0.59
	98.02	97.63	98.17	
	(69.29, 119.26)	(85.45, 114.83)	(63.61, 121.76)	
eGFR change (ml/min/1.73m^2^), median (IQR)	*N* = 97	*N* = 30	*N* = 67	0.014*
	-0.23	-12.03	5.13	
	(-31.21, 18.98)	(-33.13, 9.74)	(-14.44, 35.66)	
Number of anti-HT drugs per patient, mean ± SD	1.47 ± 0.66	1.48 ± 0.64	1.46 ± 0.67	0.89
Proteinuria, N (%)	*N* = 40	*N* = 6	*N* = 34	0.011*
	19 (47.5)	0 (0)	19 (55.9)	
ACEI monotherapy, N	13	6	7	0.11
ARB monotherapy, N	2	2	0	0.023*
CCB monotherapy, N	37	8	29	0.11

*indicate statistical significance with *P*-value less than 0.05

BMI: body mass index; SBP: systolic blood pressure; DBP: diastolic blood pressure; ACEI: angiotensin converting enzyme inhibitors; ARB: angiotensin receptor blockers; CCB: calcium channel blockers; eGFR: estimated glomerular filtration rate

### Control of HT at the follow-up visit

**Fig. 1:**
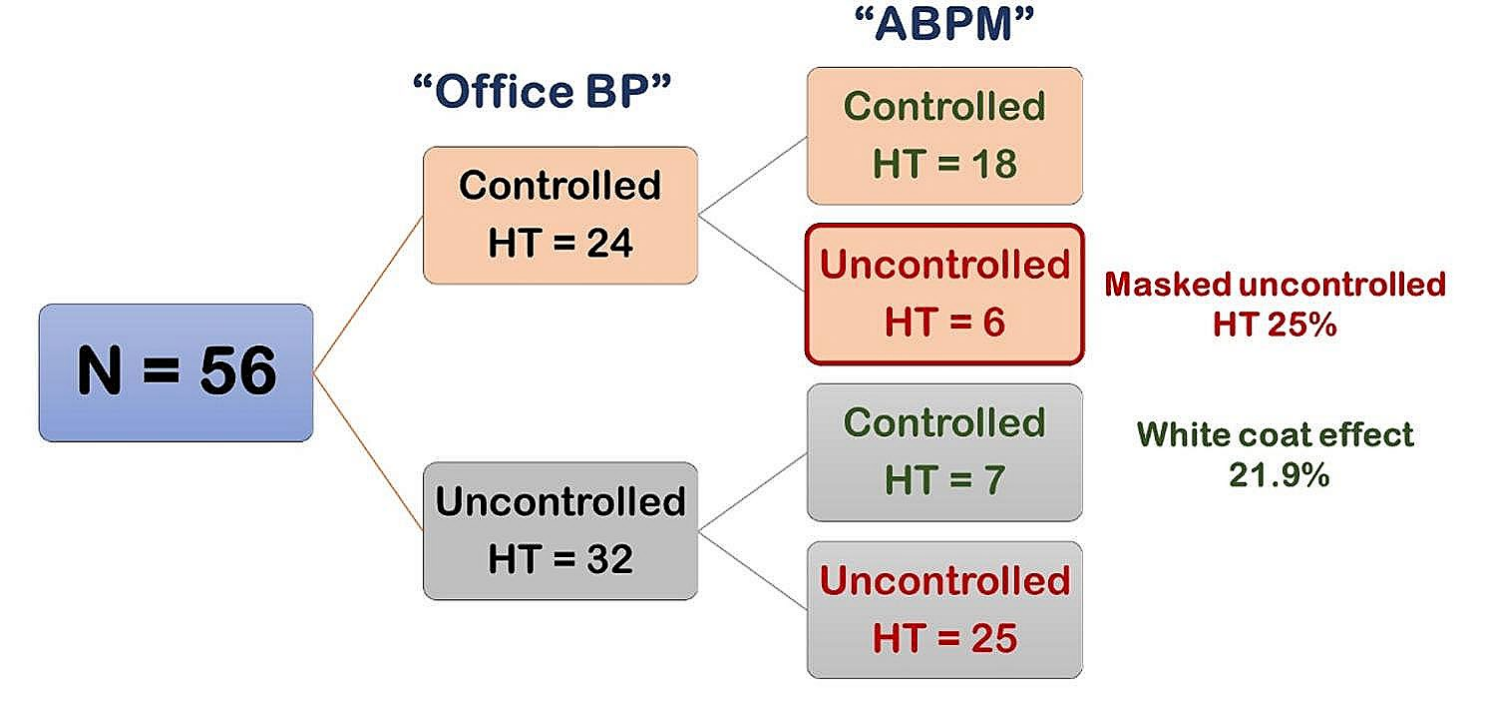
Comparison of the office BP and ABPM results in 56 primary HT patients

**Fig. 2:**
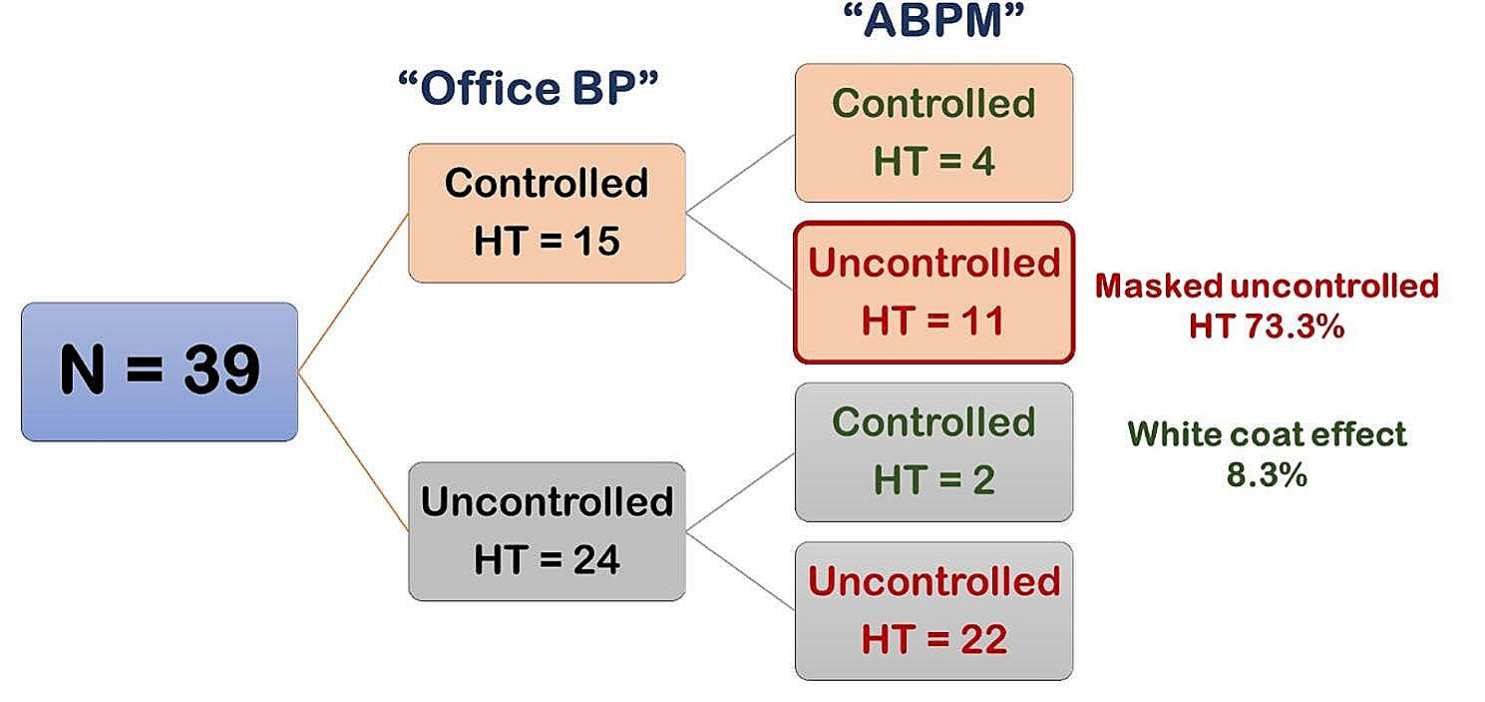
Comparison of the office BP and ABPM results in 39 secondary HT patients

Controlled HT was detected in 41.1% and 33.3% by office BP measurement and ABPM, respectively. The median (IQR) time of the first ABPM study after treatment of HT was 1.8 (0.7, 3.7) years. Based on the ABPM results, there was a greater prevalence of controlled HT in the primary HT than in the secondary HT group (44.6% vs. 21.2%, *P* = 0.01). Comparisons between the office BP and ABPM results are shown in Figs. 1 and 2. As the 2017 American Academy of Pediatrics guidelines did not provide the 75th percentile of BP, therefore thirteen patients with non-proteinuric chronic kidney disease were not assessed for controlled HT by office BP measurement. Hence, ninety-five patients had data for both office BP measurement and ABPM for comparison. Among 39 patients with controlled HT by office BP measurement, 17 patients had uncontrolled HT by ABPM (6 patients in the primary HT group and 11 patients in the secondary HT group) so called “masked uncontrolled HT”. It revealed a greater proportion of masked uncontrolled HT in the secondary HT than in the primary HT group (73.3% vs. 25%) while “white coat effect” was more prevalent in the primary HT than the secondary HT group (21.9% vs. 8.3%). The ABPM parameters results between primary and secondary HT groups are shown in Table S1.

**Fig. 3:**
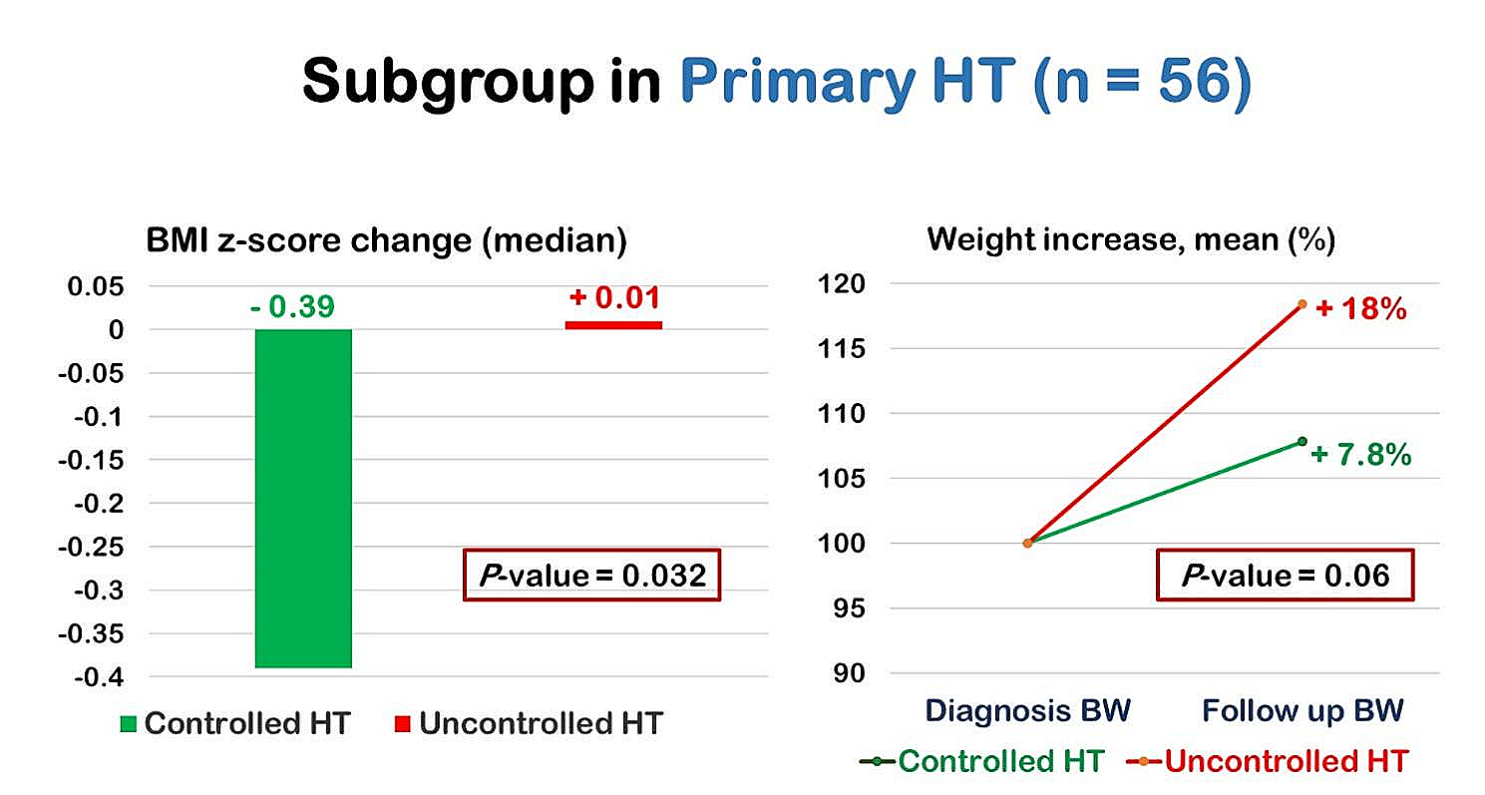
BMI z-score change and weight increase in 56 primary HT patients with controlled HT and uncontrolled HT assessed by ABPM

Control of HT among 56 patients with primary HT and 44 patients with kidney causes of HT are presented in Table 2 and S2, respectively. Among fifty-six primary HT patients, the median (IQR) BMI z-score change at the follow-up visit from the diagnosis visit was significantly decreased in the controlled HT group than the uncontrolled HT group [-0.39 (-0.57, 0.12) vs. 0.01 (-0.39, 0.22), *P* = 0.032]. In addition, the mean percentage of increase in body weight had a trend to be greater in the uncontrolled HT than in the controlled HT group (18% vs. 7.8%, *P* = 0.06) as shown in Fig. 3. Multivariate logistic regression revealed that primary HT was significantly associated with a lower risk of uncontrolled HT (Table 3).

**Table 2:** Demographic data at diagnosis and follow-up in primary HT children with controlled and uncontrolled HT

Parameters	All patients (*N* = 56)	Controlled HT (*N* = 25)	Uncontrolled HT (*N* = 31)	*P* value
**At diagnosis**				
Age, y (mean ± SD)	12.2 ± 3.67	13.09 ± 3.22	11.48 ± 3.9	0.11
Male, N (%)	40 (71.4)	19 (76)	21 (67.7)	0.56
Diagnosis BMI z-score, median (IQR)	2.5 (1.45, 2.86)	2.51 (1.43, 2.78)	2.5 (1.35, 3.18)	0.79
Office SBP index, median (IQR)	1.04 (1, 1.08)	1.04 (1, 1.09)	1.03 (1, 1.08)	0.66
Office DBP index, median (IQR)	0.96 (0.91, 1.04)	0.97 (0.9, 1.05)	0.96 (0.91, 1.04)	0.81
eGFR (ml/min/1.73m^2^), mean ± SD	*N* = 52 108.89 ± 27.05	*N* = 24 103.3 ± 30.12	*N* = 28 113.68 ± 23.62	0.17
**At Follow-up**				
Age, y (mean ± SD)	13.91 ± 3.86	14.72 ± 3.34	13.25 ± 4.17	0.16
The 1st ABPM after treating HT (y), median (IQR)	1 (0.5, 2.18)	1.11 (0.5, 2.5)	0.9 (0.4, 2)	0.62
BMI z score, mean ± SD	2.13 ± 1.56	1.96 ± 1.06	2.26 ± 1.87	0.49
BMI z score change, median (IQR)	-0.14 (-0.51, 0.14)	-0.39 (-0.57, 0.12)	0.01 (-0.39, 0.22)	0.032*
24-hr ABPM SBP index, mean ± SD	0.98 ± 0.08	0.92 ± 0.05	1.04 ± 0.06	**< 0.001**
24-hr ABPM DBP index, mean ± SD	0.89 ± 0.08	0.85 ± 0.05	0.93 ± 0.07	**< 0.001**
eGFR (ml/min/1.73m^2^), median (IQR)	*N* = 49 106.49 (90.94,120.32)	*N* = 19 105.94 (84.37, 119.69)	*N* = 30 108.06 (91.57, 122.81)	0.4
eGFR change (ml/min/1.73m^2^), median (IQR)	*N* = 45 0.36 (-17.46, 9.52)	*N* = 19 -5.18 (-22.01, 11.76)	*N* = 26 1.23 (-15.62, 9.05)	0.37
Number of anti-HT drugs per patient, mean ± SD	*N* = 41 1.34 ± 0.53	*N* = 17 1.35 ± 0.49	*N* = 24 1.33 ± 0.57	0.91
Proteinuria, N (%)	*N* = 7 1 (14.29)	*N* = 3 0 (0)	*N* = 4 1 (25)	0.35
ACEI monotherapy, N	7	4	3	0.26
ARB monotherapy, N	2	2	0	0.07
CCB monotherapy, N	15	5	10	0.49

*indicate statistical significance with *P*-value less than 0.05

BMI: body mass index; SBP: systolic blood pressure; DBP: diastolic blood pressure; ACEI: angiotensin converting enzyme inhibitors; ARB: angiotensin receptor blockers; CCB: calcium channel blockers; eGFR: estimated glomerular filtration rate

**Table 3:** Multivariate analysis of parameters associated with uncontrolled HT

Parameters	Exp(ß) (95%CI)	*p*-value
Primary HT	0.13 (0.02–0.81)	0.029*
Age at diagnosis	0.93 (0.81–1.07)	0.297
Male	0.93 (0.33–2.63)	0.896
eGFR at diagnosis	0.98 (0.96–1.01)	0.149
BMI z-score at follow-up	1.11 (0.81–1.53)	0.516
eGFR change	0.98 (0.95–1.01)	0.107

*indicate statistical significance with *P*-value less than 0.05

HT: hypertension; eGFR: estimated glomerular filtration rate; BMI: body mass index

### Control of HT and LVH

Sixty-eight patients (63%) had available echocardiographic results as shown in Table 4. The median (IQR) time between echocardiography and ABPM study was 6.75 (1.71, 13.64) months. The total prevalence of LVH was detected in 18 patients. Although not significant, there were a more proportion of LVH in the uncontrolled HT than the controlled HT group (29.79% vs. 19.05%). Interestingly, the uncontrolled HT group with LVH had a significantly longer duration of HT than those without LVH (3.6 vs. 1.1 years, *P* = 0.016). In addition, diagnosis of uncontrolled HT by ABPM showed a better sensitivity (77.8% vs. 55.8%) and a better negative predictive value (80.9% vs. 70.8%) to predict LVH than those of the office BP measurement.

**Table 4:** Data of 68 patients with echocardiographic results compared between the controlled and uncontrolled HT groups assessed by ABPM

Parameters	All	Controlled HT	Uncontrolled HT	*P* value
LVH, N (%)	*N* = 68	*N* = 21	*N* = 47	
	18 (26.5)	4 (19.1)	14 (29.8)	0.35
LVMI (g/m^2^), median (IQR)				
- All	*N* = 68	*N* = 21	*N* = 47	
	93.1 (76.8, 106.8)	90.4 (83.2, 102.5)	93.89 (76.4, 111)	0.85
- Male	*N* = 45	*N* = 14	*N* = 31	
	100 (84.9, 115)	95.5 (80.6, 112.5)	100.7 (85.5, 116)	0.7
- Female	*N* = 23	*N* = 7	*N* = 16	
	84 (71, 95)	87 (84, 92.9)	77.15 (65.8, 98)	0.42

LVH: left ventricular hypertrophy; LVMI: left ventricular mass index

## Discussion

The present study revealed that a significantly greater rate of controlled HT by ABPM was detected in the primary HT than the secondary HT group. Primary HT patients with controlled HT by ABPM had a significant decrease in BMI z-score compared to those with uncontrolled HT. In addition, among patients with uncontrolled HT by ABPM, patients with LVH had a significantly longer duration of HT than those without LVH.

Previous studies reported the prevalence of uncontrolled HT ranging from 23 to 43.6% by office BP measurement and 47–78% by ABPM [4–6]. This was consistent with the present study showing the prevalence of uncontrolled HT at 58.9% and 66.7%, by office BP measurement and ABPM, respectively. Seeman et al. reported that masked uncontrolled HT and white coat effect were detected in 23% (45/195) and 13% (25/195) compared with 17.9% (17/95) and 9.5% (9/95) in the present study [4]. Moreover, the discordant results between office BP measurement and ABPM in the present study were also detected more common in patients with secondary HT than primary HT (33.3% vs. 23.2%). This finding supported the practice point recommended by the Kidney Disease Improving Global Outcomes (KDIGO) 2021 guidelines [14] suggesting monitoring BP with ABPM once a year in children with chronic kidney disease.

In the present study, patients with primary HT had a higher rate of controlled HT than that of patients with secondary HT. In contrast, Silverstein et al. reported a study of 158 patients (34.4% had primary HT and 65.6% had secondary HT). They found that post-therapy office SBP and DBP were significantly lower in secondary HT patients than in primary HT patients and uncontrolled HT was detected in 80% of children with primary HT and in 58% of children with secondary HT [15]. The different BP goal and methods might be the causes of different results between the two studies. The present study used criteria recommended by the 2016 European Society of Hypertension guidelines and used ABPM for BP control assessment while their study used a fix BP goal at the 95th percentile for all groups of patients and office BP was used to assess BP control. Another study by Seeman et al. did not find any differences in BP control between children with primary and secondary HT [4]. It could be possible that the present study recruited a more proportion of patients with primary HT than that of the study by Seeman et al. (51.9% vs. 14.9%). The uncontrolled HT group also had a higher proportion of patients with proteinuria. This could be due to a higher proportion of patients with kidney cause of HT in the uncontrolled HT group.

Generally, patients with primary HT usually have high BMI and a higher BMI is positively associated with a high systolic BP [16]. Such patients with a decrease in BMI may have a good control of BP. The present study demonstrated that among 56 patients with primary HT those with controlled HT had a greater decrease in BMI z-score compared to that of those with uncontrolled HT even though they received the same instruction for lifestyle modification at the initial diagnosis. A mean percentage of increase in body weight also had a trend to be lesser in the controlled HT than in the uncontrolled HT group. Similar result was reported in a school-based study by Angelopoulos et al. showing that the intervention to decrease BMI was associated with a decrease in systolic and diastolic BP in 646 children over 12-month period [17]. An underlying mechanism in which a decrease in weight leads to a decrease in BP could be due to the fact that it decreases sympathetic nervous system activity which has a direct effect on arterial pressure (decreased peripheral vasoconstriction), an indirect effect on arterial pressure (improved pressure natriuresis resulting in lower intravascular volume), and a decrease in renin release from the kidney [18]. Altogether, these findings further support that a lifestyle modification to decrease weight or weight control plays a crucial role in the management of patients with primary HT. On the other hand, controlled HT in patients with kidney cause of HT may have a slow decline in eGFR overtime as uncontrolled HT is a risk factor of progressive damage of kidney function [19]. However, the present study did not show a slower eGFR decline in patients with kidney cause of HT who had controlled HT than those who had uncontrolled HT. It may be because most patients with kidney cause of HT had a low eGFR at the diagnosis and later had an improve in kidney function after treatment of HT.

LVH, an increase in left ventricular mass (LVM) in response to HT, is currently a well-known pediatric surrogate marker for HT-induced morbidity and mortality in adults [20]. Pediatric definition of LVH and abnormal LVM is not uniformed and it varies between studies. The present study used the definition from the 2017 AAP guidelines [1]. It was noted that about one-third of patients who had available echocardiographic data had LVH. The result showed that patients with uncontrolled HT and LVH had a longer duration of HT than that of those without LVH (3.6 vs. 1.1 years, respectively). However, this could not demonstrate whether LVH was a result of uncontrolled HT in these patients as no echocardiographic studies were performed at the diagnosis of HT; and the exact duration of HT was not known in both groups as we did not know how long these children had been hypertensive before the diagnosis was made.

There are several limitations to our study. Firstly, there was a small number of patients enrolled with only 20% underwent ABPM at the diagnosis of HT. Secondly, office BP was measured with a non-validated device and the average of two instead of three measurements were used as office BP value in each patient. Thirdly, echocardiographic data were not available in 40 patients (38%). Fourthly, there were no available data for the 75th percentile in the AAP 2017 office BP table leading to 13 patients (12%) without a comparison between office BP and ABPM. Finally, adherence to anti-HT medications and their effects on BP control were not evaluated due to its retrospective design with only 83% of patients received anti-HT medications. A large, prospective cohort of hypertensive children with information regarding adherence to medications and end-organ damages is needed to explore the factors associated with control of BP.

In conclusion, by using ABPM assessment, only one-third of patients had controlled HT and having primary HT was negatively associated with uncontrolled HT. The uncontrolled HT group with a long duration of HT was at risk of LVH. Therefore, ABPM is a useful method for evaluation of BP control in hypertensive children and adolescents especially in the secondary HT group with a high rate of masked uncontrolled HT to early detect hypertensive end-organ damage.

### Electronic supplementary material

Below is the link to the electronic supplementary material.

Supplementary Material 1

## Data Availability

The datasets used and/or analyzed during the present study available from the corresponding author on reasonable request.
