# Supplementary material for: Control of blood pressure in hypertensive children and adolescents assessed by ambulatory blood pressure monitoring
Source: BMC Pediatr. 2024 Apr 25;24:269. doi: 10.1186/s12887-024-04732-z (PMC11044297; doi:10.1186/s12887-024-04732-z)
Supplement: Supplementary file 1 — Supplementary Material 1 [file 12887_2024_4732_MOESM1_ESM.docx]

**Supplemental Table S1** Comparison of ABPM parameters between patients with primary and secondary HT

| Parameters | Primary HT  (N = 56) | Secondary HT  (N = 52) | *P*  value |
| --- | --- | --- | --- |
| 24-hr SBP index, mean $\boldsymbol{\pm}$ SD | 0.98 ± 0.08 | 1.02 ± 0.08 | **0.048*** |
| 24-hr DBP index, median (IQR) | 0.88 (0.83,0.95) | 0.98 (0.92,1.08) | **<0.001*** |
| Daytime SBP index, mean $\boldsymbol{\pm}$ SD | 0.97 ± 0.09 | 1 ± 0.09 | 0.14 |
| Daytime DBP index, median (IQR) | 0.86 (0.82,0.91) | 0.93 (0.88,1.03) | **<0.001*** |
| Nighttime SBP index, median (IQR) | 0.96 (0.91,1.04) | 1.04 (0.96,1.14) | **<0.001*** |
| Nighttime DBP index, median (IQR) | 0.92 (0.85,1) | 1.08 (0.97,1.19) | **<0.001*** |
| SBP dipping (%), mean ± SD | 11.94 ± 7.96 | 7.96 ± 6.84 | **0.003*** |
| DBP dipping (%), mean ± SD | 14.24 ± 6.81 | 10.81 ± 8.24 | **0.02*** |

*indicate statistical significance with *P*-value less than 0.05

SBP, systolic blood pressure; DBP, diastolic blood pressure

**Supplemental Table S2** Demographic data at diagnosis and follow-up in 44 patients with kidney cause of HT

| Parameters | All patients  (N = 44) | Controlled HT  (N = 7) | Uncontrolled HT (N = 37) | *P*  value |
| --- | --- | --- | --- | --- |
| At diagnosis | | | | |
| Age, y (mean ± SD) | 11.04 ± 4.26 | 7 ± 4.56 | 11.82 ± 3.8 | 0.05 |
| Male, N (%) | 26 (59.1) | 3 (42.9) | 23 (62.2) | 0.42 |
| Diagnosis BMI z-score, median (IQR) | -0.14  (-1.13, 0.81) | 0.78  (-0.8, 3.32) | -0.34  (-1.26, 0.66) | 0.07 |
| Office SBP index, median (IQR) | 1.03 (0.99, 1.1) | 1.03 (0.96, 1.06) | 1.03 (0.99, 1.11) | 0.683 |
| Office DBP index, median (IQR) | 1.03 (0.95, 1.13) | 1.13 (1.03, 1.44) | 1.01 (0.93, 1.1) | **0.017*** |
| eGFR (ml/min/1.73m^2^), mean ± SD | 47.83 ± 40.14 | 54.43 ± 38.92 | 46.59 ± 40.77 | 0.641 |
| At Follow-up | | | | |
| Age, y (mean ± SD) | 14.82 ± 3.63 | 12.18 ± 4.58 | 15.32 ± 3.25 | **0.03*** |
| The 1^st^ ABPM after treating HT (y), median (IQR) | 3.1 (1.1, 5.45) | 5.7 (2.3, 8.2) | 3.1 (1.05, 4.4) | 0.172 |
| BMI z score, mean ± SD | 0.01 $\pm$ 1.44 | 0.92 $\pm$ 0.99 | -0.16 $\pm$ 1.46 | 0.07 |
| BMI z score change, median (IQR) | 0.06  (-1, 0.73) | 0.28  (-1.47, 0.91) | 0.06  (-0.96, 0.72) | 0.736 |
| 24-hr ABPM SBP index, mean ± SD | 1.03 ± 0.08 | 0.92 ± 0.05 | 1.05 ± 0.07 | <0.001* |
| 24-hr ABPM DBP index,  median (IQR) | 1.01 (0.93, 1.09) | 0.9 (0.82, 0.93) | 1.03 (0.96,1.12) | <0.001* |
| eGFR (ml/min/1.73m^2^), median (IQR) | 64.48  (52.86, 84.2) | 63.83  (51.72, 67.6) | 66.25  (53.03, 87.12) | 0.619 |
| eGFR change (ml/min/1.73m^2^), median (IQR) | 17.02  (-2.07, 55.52) | -2.56  (-7.37, 56.41) | 19.7  (1.11, 55.84) | 0.153 |
| Number of anti-HT drugs per patient, mean ± SD | N = 41  1.51 ± 0.71 | N = 6  1.33 ± 0.82 | N = 35  1.54 ± 0.7 | 0.512 |
| Proteinuria, N (%) | N = 31  18 (58.06) | N = 2  0 (0) | N = 29  18 (62.07) | 0.085 |
| ACEI monotherapy, N | 6 | 2 | 4 | 0.349 |
| ARB monotherapy, N | 0 | 0 | 0 | NA |
| CCB monotherapy, N | 19 | 3 | 16 | 0.349 |

*indicate statistical significance with *P*-value less than 0.05

BMI, body mass index; SBP, systolic blood pressure; DBP, diastolic blood pressure; ACEI, angiotensin converting enzyme inhibitors; ARB, angiotensin receptor blockers; CCB, calcium channel blockers; eGFR, estimated glomerular filtration rate; ABPM, ambulatory blood pressure monitoring; NA, not applicable.
